# Supplementary material for: Biallelic mutations in valyl-tRNA synthetase gene VARS are associated with a progressive neurodevelopmental epileptic encephalopathy
Source: Nat Commun. 2019 Feb 12;10:707. doi: 10.1038/s41467-018-07067-3 (PMC6372641; doi:10.1038/s41467-018-07067-3)
Supplement: Supplementary file 1 — Supplementary Information [file 41467_2018_7067_MOESM1_ESM.pdf]

Supplementary Information:

Biallelic mutations in valyl-tRNA synthetase gene *VARS* are associated with a progressive neurodevelopmental epileptic encephalopathy

Friedman et al

Supplementary Figure 1

Supplementary Figure 1a

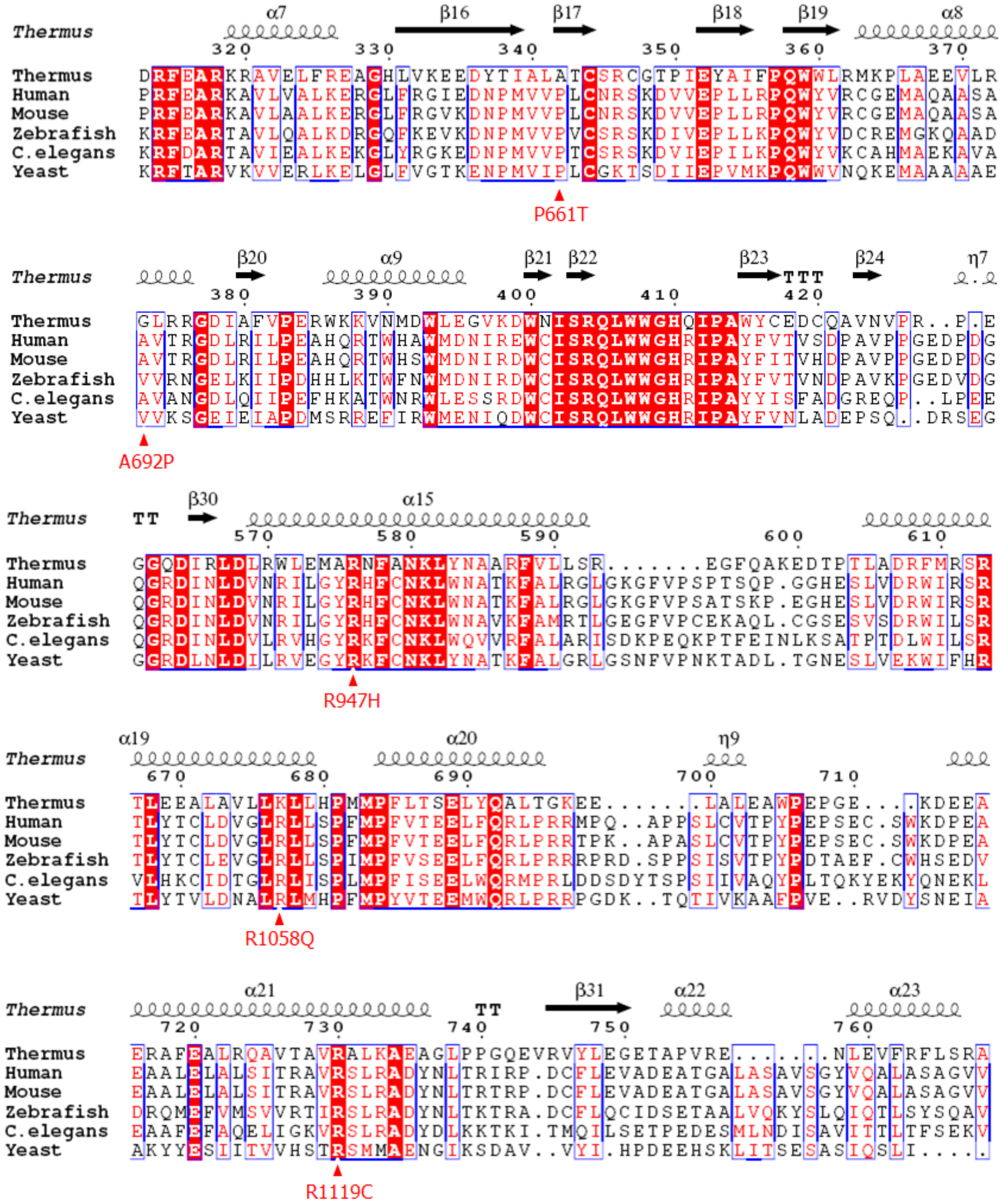

Supplementary Figure 1b

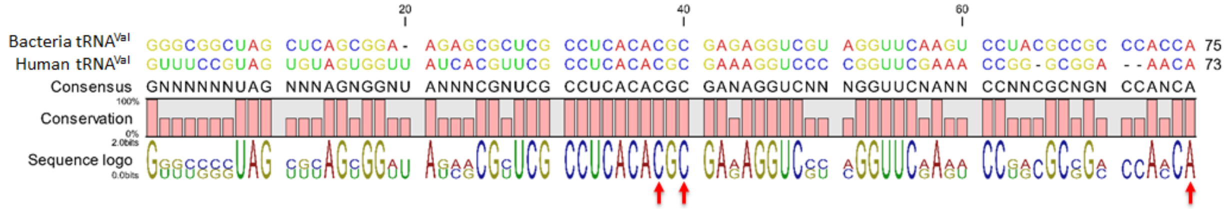

Supplementary Figure 1. Alignments of VARS protein sequences and tRNA^{Val} nucleotide sequences. (**a**) Protein alignment of VARS of several species including Thermus (*T. thermophilus*), Human (*H. sapiens*), Mouse (*M. musculus*), Zebrafish (*D. rerio*), C. elegans, and Yeast (*S. cerevisiae*). Multiple sequence alignment was performed by ClustalW. Secondary structure elements were displayed above the sequences by Espript 3 [1], using structural templates of *T. thermophilus* VARS structure (PDB: 1GAX). Shown is the partial alignment encompassing the mutational sites (indicated by arrows below the sequences). The numbering of amino acids is based on the *T. thermophilus* sequence. Numbering according to the human mutated residue is below i.e. *T. thermophilus* residue 342 is equivalent to *H. sapiens* residue 661. Boxes represent the regions of highest conservation. (**b**) Nucleotide sequence alignment of human and Thermus tRNA^{Val}. The Thermus tRNA^{Val} sequence from PDB (1GAX) and one representative human tRNA^{Val} sequence from the Genomic tRNA Database (http://gtrnadb.ucsc.edu/) were used for alignment. Nucleotides C-37, C-39 and A-75 (red arrows) are conserved, and also conserved in most of the human tRNA^{Val} sequences found in the database.

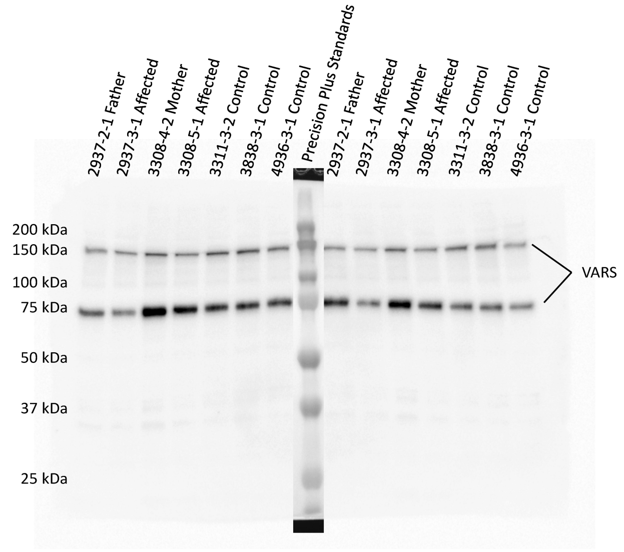

PVDF membrane probed with rabbit polyclonal anti-VARS (Abclonal A4182; 1:1000)

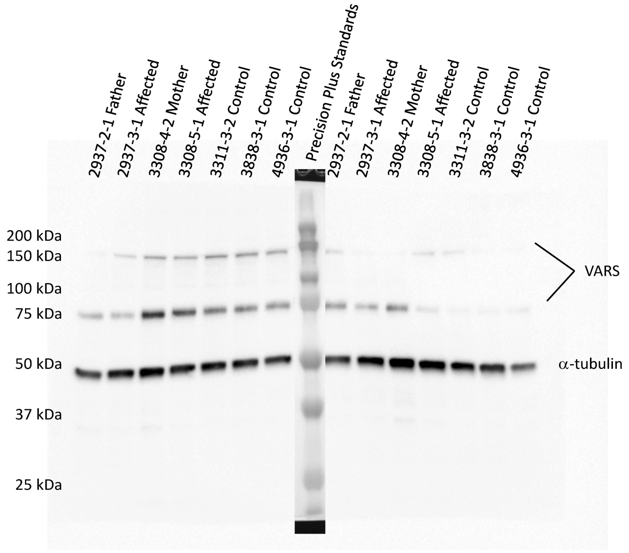

Same membrane re-probed with mouse monoclonal anti-α-tubulin (Sigma T6074; 0.2 ug/ml)

Supplementary Figure 2

Western Blot showing VARS protein levels in affected patients, heterozygous parents and controls. VARS expected molecular weight 140 kDa. Protein concentration normalized to 8ug and run on 10% Criterion pre-cast gel with Precision Plus Pre-Stained Protein Dual Color Standards. Transferred to PVDF membrane. Epi-white image of protein standards cropped in.

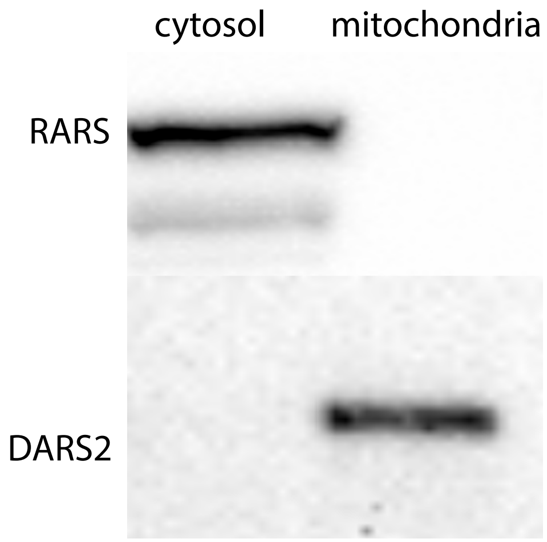

Supplementary Figure 3

Western blot of the cytosolic and mitochondria fraction of a fibroblast cell line. Cells were disrupted by three cycles of freeze-thaw. After centrifugation, the supernatant was loaded on a 12% stain-free SDS gel (Bio Rad) (first lane). The pellet was resuspended in a buffer containing SDS and was loaded (second lane). Proteins were transferred to a PVDF membrane using the Transblot system (Bio Rad). After 1 hour bloking in 4% milk, the membrane was incubated with anti-DARS2 antibody (HPA26528, Dilution 1:2000, Sigma, Deiselhofen, Germany) and anti-RARS (13434687, 1:3000, Fisher Scienitific, Landsmeer, The Netherlands). Anti-rabbit antibody (PO448, 1:10.000, Dako, Glostrup, Denmark) was used as secondary antibody. Immune complexes were detected by enhanced chemiluminescence (Lumilight Plus), according to the manufacturer’s specifications (Roche, Indianapolis, IN, USA).

Supplementary Note

Detailed Sequencing Variant Filtering and Prioritization

Patient samples were evaluated in three independent laboratories.

Family 3007

Blood was drawn immediately following consent for trio whole genome sequencing. DNA was subsequently extracted and sequenced on a HiSeq 4000 (Illumina) with paired 151-nt reads. Rapid alignment and nucleotide variant calling was performed using the Dragen (Edico Genome, San Diego, CA) hardware and software[2]. Yield was 126 Gb, 139 Gb, and 148Gb for the proband, mother, and father, respectively. This resulted in 4,842,119, 4,747,783 and 4,654,791 distinct variant calls, respectively. Variants were annotated and analyzed in Opal Clinical (Fabric Genomics, Oakland, CA)[3]. Opal system uses a variety of effect prediction programs in aggregate to establish an Omicia Score (e.g. SIFT, PolyPhen, VEP, SNAP2, MutationTaster) for interpretation of their results, 0-1, closer to 1 as most damaging. Variants were filtered to retain those in coding and splice site regions and those with allele frequencies of <0.5% in the Exome Variant Server, 1000 Genomes Samples, and Exome Aggregation Consortium database[4], resulting in 1282 proband calls. A custom, patient-specific gene panel was built in Phenolyzer[5] using Human Phenotype Ontology (HPO)[6.] This panel included 1282 genes related to the following HPO terms: Global Developmental Delay (HP:0001263), Elevated Hepatic Transaminases (HP:0002910), and Infantile Spasms (HP:0012469). Variants were further filtered to retain those mapping to these 1298 genes yielding 91 proband calls. Manual curation of the 1292 variant calls, both filtered and non-filtered by gene panel, was performed according to ACMG guidelines[7]. Five variants were selected for further consideration and are included in Supplemental Table 1. All calls except the homozygous variants in *VARS* were discarded due to poor phenotypic overlap (*TDP2, SCO2, ITGA7 and KIF1A*), failure to find a second variant for an autosomal recessive condition (*TDP2, SCO2, ITGA7*) or variant inherited from an unaffected parent in an autosomal dominant condition (*KIF1A*). *VARS* variants were initially considered suspicious but of uncertain significance due to only a single report implicating the *VARS* gene in human disease in the literature. *VARS* was entered into GeneMatcher[8] leading to identification of multiple other groups (including GB31) also considering *VARS* variants in patients with similar phenotype. Identification of additional cases (2937, 3308, and 3439) resulted from communication through previous collaborative network with laboratory evaluating these families.

Family GB31

Genomic DNA was extracted from peripheral blood cells using Qiagen Gentra Puregene Blood Kit (Qiagen, Hilden, Germany) according to the manufacturer's instructions. As previously published[9, 10], whole exome sequencing was performed using PerkinElmer's sequencing service using the Agilent Sure Select Human All Exon Capture V4 Kit and performed (two paired-end 100-bp reads) with the Illumina HiSeq 2000 system. Reads were aligned to the reference human genome (UCSC Genome Browser hg19) with the Genome Analysis Toolkit (GATK)[11,12] SAMtools[11,13], Picard (see web resource) and CASAVA v1.8[14] and annotated using the snpEff software tool (http://snpeff.sourceforge.net/), as well as visualization tools from PerkinElmer. Variants were filtered to less than 1% minor allele frequency in population databases, then prioritized by the suspected mode of inheritance, American College of Medical Genetics categorization, OMIM identity and *in silico* prediction tools. Candidate genes were correlated to the patient's phenotype, gene function and expression. Sanger sequencing of the patient and her parents was performed to confirm the variants and co-segregation using primer pairs designed with the primer3 software. PCR products were sequenced at the McGill University and Genome Quebec Innovation Centre using an ABI 3730xl DNA Analyzer (ABI; Applied Biosystems, Foster City, CA, USA).

Sequences were analyzed using SeqMan 4.03 (DNAStar, Wisconsin, USA). A step-wise filtering approach was used to analyze genes containing rare genetic variants (minor allele frequency less than 1%). First, a gene panel of 147 microcephaly-related genes was created using NCBI. From this list, 10 variants were identified in 10 different microcephaly-associated genes. In a second analysis, the remaining rare variants were then analyzed to identify novel pathogenic gene candidates, assuming an autosomal recessive inheritance. This analysis revealed a total of 43 potential candidate genes, including 28 genes with 2 or more heterozygous variants, and 12 genes with one homozygous variant. From these two analyses, 4 genes with most likely pathogenic variants (*CENPE, RAD50, KIF11,* and *VARS)* were further explored and validated via Sanger Sequencing (Supplemental Table 1). *CENPE* and *RAD50* were not further explored as candidates as they contained only one variant, and no second variant or a long deletion was identified, which is not compatible with an autosomal recessive condition. The variant in *KIF11,* thought to be associated with autosomal dominant inheritance, was also removed as it was previously reported in healthy individuals in gnomAD. Therefore, *VARS* remained as the final candidate, and bi-allelic inheritance was confirmed following co-segregation analysis.

Families 2937, 3308 and 3439

Blood DNA was extracted using Qiagen reagents (Qiagen Inc., USA), then subjected to exome capture with either the Agilent SureSelect Human All Exome 50 Mb kit (Agilent Technologies, Inc., USA) or the Illumina Rapid Capture 37 Mb Enrichment kit. Sequencing with 100-bp paired-end reads was performed using either the Illumina HiSeq2000 or HiSeq4000 instruments (Illumina, Inc., USA), resulting in >94% recovery at 10× coverage and >85% recovery at 20x coverage. GATK best practices pipeline was used for SNP and INDEL variant identification (http://www.broadinstitute.org/gatk/). Variants were annotated with in-house software[15] and homozygous variant prioritization was done using custom Python scripts (available upon request) to keep variants with MAF <0.001 in our sequenced cohort, or with high scores for likelihood to damage protein function. Sanger sequencing was used for segregation testing in all available family members. Variants selected for consideration are listed in Supplemental Table 1.

Supplementary References

1. Robert X., Gouet P. Deciphering key features in protein structures with the new ENDscript server. *Nucleic Acids Res* **42**, W320-324 (2014).

2. Miller, N.A. et al. A 26-hour system of highly sensitive whole genome sequencing for emergency management of genetic diseases. *Genome Med.* **7:**100 (2015).

3. Coonrod, E.M., Margraf, R.L., Russell A., Voelkerding K.V., Reese M.G. Clinical analysis of genome next-generation sequencing data using the Omicia platform. *Expert Rev Mol Diagn*. **13**(6):529-40 (2013).

4. Karczewski et al. Analysis of protein-coding genetic variation in 60,706 humans*. Nature* **536**(7616):285-91 (2014).

5.Yang, H., Robinson, P.N., and Wang, K. Phenolyzer: phenotype-based prioritization of candidate genes for human diseases. Nat Methods. 12(9):841-3 (2015).

6. Köhler, S. et al. The human phenotype ontology in 2017. *Nucleic Acids Res* **45** (2017).

7. Richards, S. et al. Standards and Guidelines for the Interpretation of Sequence Variants: A Joint Consensus Recommendation of the American College of Medical Genetics and Genomics and the Association for Molecular Pathology *Genet Med.* **17**(5): 405–424 (2015),

8. Sobreira, N., Schiettecatte F., Valle D., Hamosh A. GeneMatcher: a matching tool for connecting investigators with an interest in the same gene. *Hum Mutat.* **36**(10):928-30 (2015).

9.Mendes, M.I. et al., Bi-allelic mutations in EPRS, encoding the glutamyl-prolyl-aminoacyl-tRNA synthetase cause a hypomyelinating leukodystrophy, *Am J Hum Genet,* 102(4):676-684 (2018).

10. Thiffault, I. et al., Recessive mutations in POLR1C cause a leukodystrophy by impairing biogenesis of RNA polymerase III. *Nature Comm* July 7;**6:**7623 (2015).

11. DePristo, M. A. et al. A framework for variation discovery and genotyping using next-generation DNA sequencing data. *Nat. Genet.* **43**;491–498 (2011).

12. McKenna, A. et al. The Genome Analysis Toolkit: a MapReduce framework for analyzing next-generation DNA sequencing data. *Genome Res.* **20**;1297–1303 (2010).

13. Li, H. et al. The sequence alignment/map format and SAMtools. *Bioinformatics* **25**;2078–2079 (2009).

14. Cheng, A. Y., Teo, Y. Y. & Ong, R. T. Assessing single nucleotide variant detection and genotype calling on whole-genome sequenced individuals. *Bioinformatics* **30**;1707–1713 (2014).

15. T. J. Dixon-Salazar, T.J et al. Exome sequencing can improve diagnosis and alter patient management. Sci. *Transl. Med.* 4, 138ra78 (2012).
