# Supplementary material for: Biallelic mutations in valyl-tRNA synthetase gene VARS are associated with a progressive neurodevelopmental epileptic encephalopathy
Source: Nat Commun. 2019 Feb 12;10:707. doi: 10.1038/s41467-018-07067-3 (PMC6372641; doi:10.1038/s41467-018-07067-3)
Supplement: Supplementary file 3 — Description of Additional Supplementary Files [file 41467_2018_7067_MOESM3_ESM.pdf]

Description of Additional Supplementary Files

File Name: Supplementary Data 1

Description: Prioritized VariantsTable lists prioritized variants for each case including allele frequency, conservation and in silico predictions. Annovar program was used for in silico predictions1 . MutationTaster: 0-1, values closer to 1 higher confidence that alteration is disease causing; MutationTaster_converted_rankscore: The rankscore is the ratio of the rank of the score over the total number of MTnew scores in dbNSFP. The scores range from 0.0931 to 0.80722. MutationTaster_pred: MutationTaster prediction,"D" ("disease_causing"), "N" ("polymorphism") The score cutoff between "D" and "N" is 0.5 for MTori and 0.328 for the rankscore; Polyphen-2: 0-1, values closer to 1 predicted more damaging substitution; Polyphen2_HDIV_rankscore: The rankscore is the ratio of the rank the score over the total number of the scores in dbNSFP; Polyphen2_HDIV_pred: Polyphen2 prediction based on HumDiv, "D" ("porobably damaging", HDIV score in [0.957,1] or rankscore in [0.52996,0.89917]), "P" ("possibly damaging", HDIV score in [0.453,0.956] or rankscore in [0.34412,0.52842]) and "B" ("benign", score in [0,0.452] or rankscore in [0.02656,0.34399]); SIFT score: 0-1, values closer to 0 indicate damaging variants; SIFT_converted_rankscore: . The rankscore is the ratio of the rank the SIFTnew score over the total number of SIFTnew 5 scores in dbNSFP; SIFT_pred: If SIFTori is smaller than 0.05 (rankscore>0.55) the corresponding NS is predicted as "D(amaging)"; otherwise it is predicted as "T(olerated)"; FATHMM_score: FATHMM default score (weighted for human inheriteddisease mutations with Disease Ontology) (FATHMMori). Scores range from -18.09 to 11.0; Fathmm Rank: FATHMMori scores were ranked among all FATHMMori scores in dbNSFP. The rankscore is the ratio of the rank of the score over the total number of FATHMMori scores in dbNSFP; FATHMM_pred: If a FATHMMori score is <=-1.5 (or rankscore <=0.81415) the corresponding NS is predicted as "D(AMAGING)"; otherwise it is predicted as "T(OLERATED)"; CADD: CADD raw score, the larger the number the more likely damaging; CADD_converted_rankscore: . The rankscore is the ratio of the rank the CADDnew score over the total number of CADDnew scores in dbNSFP; MCAP: score of above 0.025 are marked as possibly pathogenic while those below 0.025 are marked likely benign; M-CAP_converted_rankscore: . The rankscore is the ratio of the rank the M-CAPnew score over the total number of M-CAPnew scores in dbNSFP; M-CAP_pred: is predicted as "D(amaging)"; otherwise it is predicted as "T(olerated)"; phyloP-vertebrate: -11.764 to +6.24, positive scores indicate conservation, negative scores fast-evolution; n/a - Not Available.
